# Polycyclic Aromatic Hydrocarbon Concentration Levels on the Korean Peninsula between 2006 and 2008

**DOI:** 10.1100/tsw.2010.5

**Published:** 2010-01-08

**Authors:** Hang Thi Nguyen, Ki-Hyun Kim, C.-J. Ma, J.-M. Oh

**Affiliations:** ^1^ Department of Earth and Environmental Sciences, Sejong University Seoul, Republic of Korea; ^2^ Department of Environmental Science, Fukuoka Women's University, Japan; ^3^ College of Environment and Applied Chemistry, Kyunghee University, Yongin, Republic of Korea

**Keywords:** polycyclic aromatic hydrocarbons, Korea, particle phase, gaseous phase

## Abstract

Concentrations of seven polycyclic aromatic hydrocarbon (PAH) compounds — benzo(a)anthracene (BaA), chrysene (CHRY), benzo(b)fluoranthene (BbF), benzo(k)fluoranthene (BkF), dibenz(a,h)anthracene (DahA), indeno(1,2,3-cd)pyrene (I123P), and benzo(a)pyrene (BaP) — in air were measured as the sum of gas and particle fractions at 32 monitoring stations dispersed across Korea during a 2-year period (February 2006 to January 2008). The data sets were collected at intervals of 1 day (24 h) per month from each monitoring station. According to our analysis, the spatial distribution of PAH is distinguished by manmade activities between different land use types. Evaluation of total PAH (T-PAH) concentration levels, which were derived by summing up all individual compounds, revealed that the T-PAH value varied on the order of commercial (4.85 ± 4.40 ng m^-3^) rural (4.42 ± 2.73 ng m^-3^), industrial (4.27 ± 1.79 ng m^-3^), greenland (3.09 ± 3.86 ng m^-3^), and background (2.60 ± 2.54 ng m^-3^) areas. The PAH values, when compared across seasons, tend to peak consistently during the winter (or spring) due to the active consumption of fossil fuels. The overall results of this study confirm that the pollution status of PAH compounds are clearly discernible not only between areas with different levels of anthropogenic activities, but also between periods with changes in environmental conditions.

## INTRODUCTION

Polycyclic aromatic hydrocarbons (PAHs) belong to one of the major toxic pollutants in air for their carcinoginicity or mutagenicity[1,2,3]. Most human carcinogenic PAHs are associated with suspended particulate matter, accounting for ~55% of the total PAH levels in aerosols[4]. PAHs are released into the atmosphere from both natural and anthropogenic sources through incomplete combustion or high-temperature pyrolytic processes of fossil fuels (and other organic materials). The global atmospheric emissions of the 16 PAHs were estimated to be 520 Gg in 2004; Asian countries generally rank the highest in emissions (55% with 290 Gg), while those of North America and Europe are less significant, covering 8.0 and 9.5%, respectively[5]. In fact, the emissions of PAHs in Europe have been declining continually by about 47% (in 43 countries) during recent decades, e.g., 2.4 (1990) to 1.3 ton year$^{-1}$ (2003), which might not have been possible without the improvements in control technology[6].

The relative contribution of different emission sources varies greatly by the combined effects of such factors as their emission strengths, geographical location, and local climatic conditions[7]. As such, the transport, deposition, and chemical transformation of PAHs are also controlled by an interplay of such parameters[8]. The distribution of PAHs is, however, explained not only by the magnitude of emission, but also by the factors controlling its stability. The importance of the latter can be summarized as (1) physical removal by dry or wet deposition; (2) atmospheric transport and dispersion by the shift of air mass, turbulence, and convection; (3) atmospheric degradation (or conversion) through physicochemical reactions; and (4) the exchange of the gaseous and particulate phase toward the phase equilibrium[1].

In this study, the basic aspects of PAH pollution were investigated using the data sets collected from several urban locations in Korea, with an emphasis on seven PAH species: benzo(a)anthracene (BaA), chrysene (CHRY), benzo(b)fluoranthene (BbF), benzo(k)fluoranthene (BkF), dibenz(a,h)anthracene (DahA), indeno(1,2,3-cd)pyrene (I123P), and benzo(a)pyrene (BaP). Table 1 provides basic information concerning their chemical formula and structures. All the analyses were made at major stations established for the routine monitoring of hazardous air pollutants (HAPs) on the Korean peninsula. Through an application of diverse statistical analysis, we attempted to describe the pollution status of PAHs across the major locations in Korea.

## MATERIALS AND METHODS

In this work, the distribution characteristics of the PAH species were investigated as the sum of gas and particle fractions using the data sets collected from HAP monitoring stations managed by the Korean Ministry of Environment (KMOE) for the period between February 2006 and January 2008. As shown in Table 2, the PAH concentrations were measured from up to 32 monitoring stations known for a relatively large population (e.g., above a half million) or strong industrial activities (Fig. 1). The Republic of Korea (RoK) consists of nine provinces and seven metropolitan cities; note that the latter physically belong within five of the provinces. The location of the individual stations can be divided into seven metropolitan cities (SL, BS, DG, IC, DJ, GJ, and US) consisting of 15 individual stations and eight provinces (GW, GG, GN, GB, JN, JB, CN, and CB). At each individual station, the collection of particle samples was conducted at monthly intervals (sampling on 1 weekday [24 h] during the midmonth by following the standard PAH measurement protocol guided by the KMOE, which is comparable to the U.S. Environmental Protection Agency To 13 method (U.S. EPA, 1999).

At each site, high-volume air samplers were run to collect total suspended particulate (TSP) samples at a flow rate of ~500 l min$^{-1}$. These particulate phase samples were collected on prebaked quartz fiber filters (QFF: 20.3 × 25.4 cm). The QFFs were preweighed in a temperature- (4°C) and humidity-controlled room, and wrapped in aluminum foil envelopes for storage until sampling. Although instrumental settings and the associated operation conditions can differ slightly between stations, the most common setup can be generalized as follows. The concentrations of PAHs were determined by gas chromatography (GC) and a mass spectrometer (MS) in selective ion monitoring (SIM) mode (Agilent GS/MSD [7890A/59750]). Separation of individual PAHs was made by a capillary column (DB-5MS, 30 m × 0.25 mm × 0.25 µm film thickness). The mass selective detector was operated in electron impact mode (70 eV). The chromatographic conditions were as follows: injector temperature, 280°C; interface temperature, 290°C; temperature program, 80°C held for 5 min, 80–300°C at 6°C min$^{-1}$, 300°C for 5 min. The flow rate of carrier gas (helium) was maintained at 1.5 ml min$^{-1}$. All injections were split with ratio 10:1 and the volume was 1 µl. The gas phase samples were captured on 10-cm medium-density polyurethane foam (PUF). All samples were spiked with internal (and surrogate) standards containing acenaphthene-$d_{10}$, chrysene-$d_{12}$, phenanthrene-$d_{10}$, perylene-$d_{12}$ (EPA 525 PAHs Internal Std. Mix 48242). The identity and subsequent retention time of each PAH was confirmed by standards containing known concentrations of both target PAH compounds and internal standard compounds.

**TABLE 1**
**Basic Information for the Seven PAHs Measured in 15 Cities/Provinces in Korea**

| Order | Full Name | Acronym | CAS No. | Chemical Formula | Chemical Structure | MW [g mol$^{-1}$] |
|---|---|---|---|---|---|---|
| 1 | Benzo[a]anthracene | BaA | 56-55-3 | $C_{18}H_{12}$ |  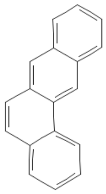 | 228.288 |
| 2 | Chrysene | CHRY | 218-01-9 | $C_{18}H_{12}$ |  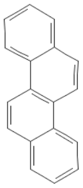 | 228.288 |
| 3 | Benzo[b]fluoranthene | BbF | 205-99-2 | $C_{20}H_{12}$ |  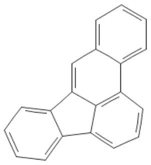 | 252.309 |
| 4 | Benzo[k]fluoranthene | BkF | 207-08-9 | $C_{20}H_{12}$ |  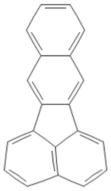 | 252.309 |
| 5 | Dibenz[a,h]anthracene | DahA | 53-70-3 | $C_{22}H_{14}$ |  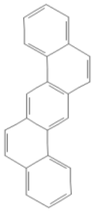 | 278.346 |
| 6 | Indeno[1,2,3-cd]pyrene | I123P | 193-39-5 | $C_{22}H_{12}$ |  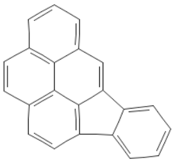 | 276.330 |
| 7 | Benzo[a]pyrene | BaP | 50-32-8 | $C_{20}H_{12}$ |  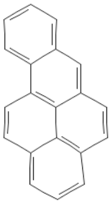 | 252.309 |

**TABLE 2**
**Acronym Codes for Individual Stations in Seven Cities (1–15) and Eight Provinces (16–32) and the Associated Information**

| Order | City (Code) | Station Code[1] | Station Name | Land Use[2] | Study Period | |
|---|---|---|---|---|---|---|
| | | | | | Start | End |
| 1 | Seoul (SL) | SL1 | Do Gok | R | 06.2 | 08.1 |
| 2 | | SL2 | Gu Ui | R | 06.2 | 08.1 |
| 3 | | SL3 | Seoul St. | C | 06.2 | 08.1 |
| 4 | Busan (BS) | BS1 | Duk Chun | R | 06.4 | 08.1 |
| 5 | | BS2 | Yeon San | C | 06.2 | 08.1 |
| 6 | Daegu (DG) | DG1 | Man Chon | R | 06.2 | 08.1 |
| 7 | | DG2 | Dae Myung | R | 06.2 | 08.1 |
| 8 | Incheon (IC) | IC1 | Soong Ui | R | 06.2 | 07.12 |
| 9 | | IC2 | Yeon Hui | C | 06.2 | 08.1 |
| 10 | | IC3 | Seok Mo | B | 06.2 | 08.1 |
| 11 | Daejeon (DJ) | DJ1 | Goo Sung | R | 06.2 | 08.1 |
| 12 | Gwangju (GJ) | GJ1 | Nong Sung | R | 06.2 | 08.1 |
| 13 | | GJ2 | Ha Nam | I | 06.2 | 08.1 |
| 14 | Ulsan (US) | US1 | Sin Jung | R | 06.2 | 08.1 |
| 15 | | US2 | Yeo Chun | I | 06.2 | 08.1 |
| 16 | Gangwon (GW) | GW1 | Shin Book | G | 06.2 | 08.1 |
| 17 | | GW2 | Bang San | B | 06.2 | 08.1 |
| 18 | Gyunggi (GG) | GG1 | Jeong Wang | I | 06.2 | 08.1 |
| 19 | | GG2 | Oh Jeon | C | 06.2 | 07.9 |
| 20 | | GG3 | Go Chun | C | 07.10 | 08.1 |
| 21 | Gyungnam (GN) | GN1 | Myung Seo | R | 06.2 | 08.1 |
| 22 | | GN2 | Bong Ahm | I | 06.2 | 08.1 |
| 23 | Gyungbuk (GB) | GB1 | Jang Heung | I | 06.2 | 08.1 |
| 24 | Junnam (JN) | JN1 | Sam Il | I | 06.2 | 08.1 |
| 25 | | JN2 | Joong | I | 06.2 | 08.1 |
| 26 | Jeonbuk (JB) | JB1 | Hyo Ja | R | 06.2 | 08.1 |
| 27 | | JB2 | So Ryong | I | 06.2 | 08.1 |
| 28 | | JB3 | Woon Ahm | I | 06.2 | 08.1 |
| 29 | Chungnam (CN) | CN1 | Won Sung | R | 06.2 | 08.1 |
| 30 | | CN2 | Dae San | I | 06.2 | 08.1 |
| 31 | | CN3 | Pa Do | B | 06.2 | 08.1 |
| 32 | Chungbuk (CB) | CB1 | Song Jeong | I | 06.2 | 08.1 |

[1] All stations are named by two capital letters of each district with the numbers.

[2] Abbreviations for land use type information: B = background, G = greenland, R = residential, C = commercial, and I = industrial.

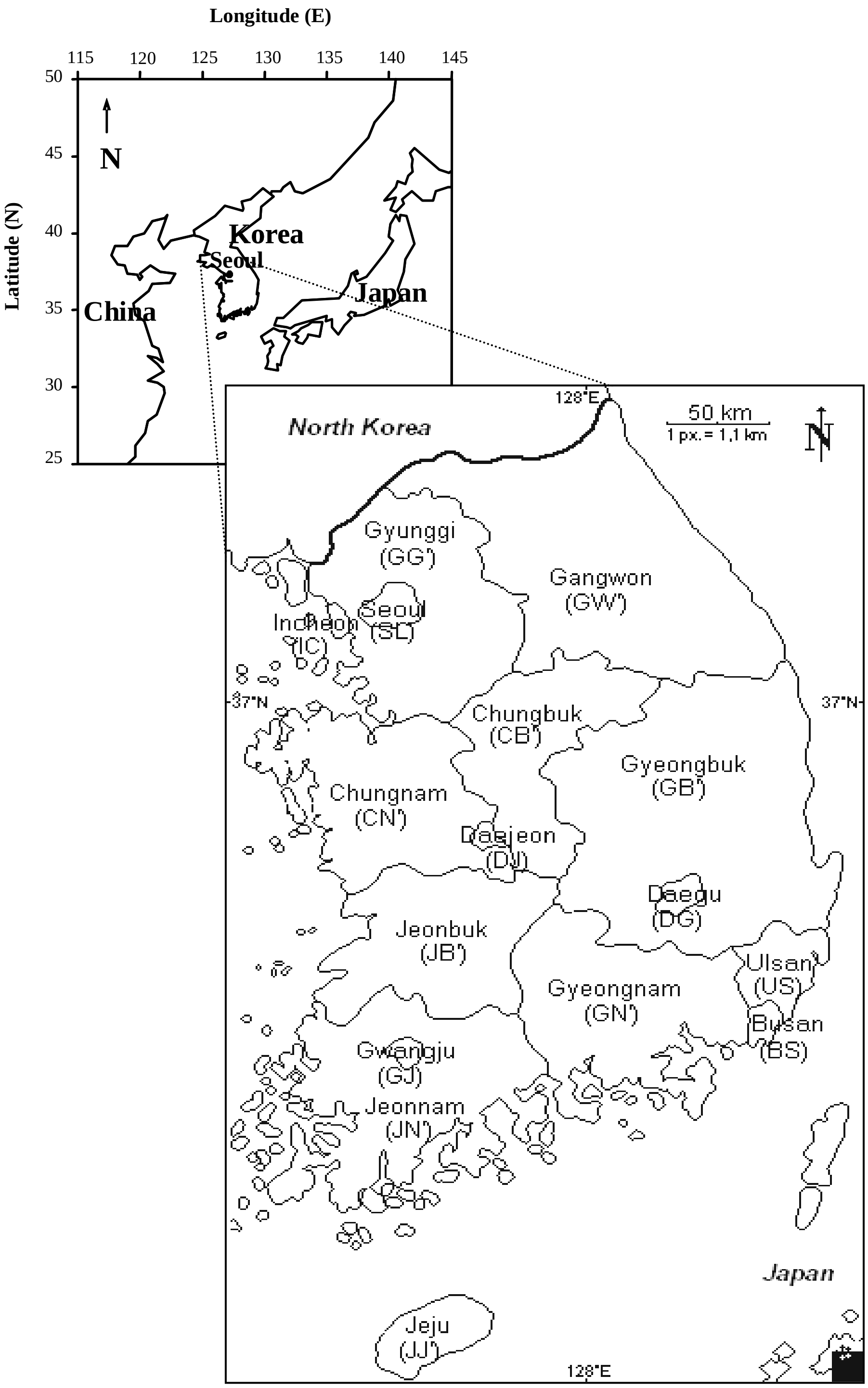

**FIGURE 1.** Geographical location of seven metropolitan cities (SL, BS, DG, IC, DJ, GJ, and US) and eight provinces (GW, GG, GN, GB, JN, JB, CN, and CB) in Korea.

Quality assurance (QA) was conducted by routine analysis of laboratory blanks, field blanks, split PUFs, and matrix spikes to reduce or eliminate the possible source of contamination or artifacts. The method detection limits, defined as 3.14 times the standard deviation (n = 7) of the low concentration standards, were in the 0.20 ng range (in absolute mass), which also corresponds to ~1 pg $m^{-3}$. These raw

PAH concentration data are then quality controlled by the procedures of KMOE and stored in its data-management network system. All the initial data sets were collected at monthly intervals from each monitoring station and stored as the sum concentration of both gaseous and particulate phases. These initial monthly data sets collected from all individual stations were then analyzed to assess the behavior of PAHs in several respects.

The acronym codes for the major districts and individual stations are shown along with the information of land use type in Table 2. It shows that 15 stations belong to seven major cites, while the remaining 17 stations are for eight provinces. As seen in Table 3, the total number of monthly measurement data for each station, while amounting up to 23, differs greatly between stations. The amount of monthly data was as low as 3 at the newly added station, like GG3, while the maximum monthly data of 23 were attained at several stations (e.g., GG1 and GW1). To allow comparison of these PAH data on a wide spatial scale, the monthly data for each station were put together to derive their representative values for seven cities and eight provinces. A comparison of relative standard error (RSE = SE × 100/mean) values of given PAH data at each city (or province) scale shows that their values within a given district boundary tend to vary in a fairly consistent manner. Hence, unless otherwise specified, the environmental behavior of all PAH species examined in this study is evaluated by the mean values for a given city (or province) at monthly intervals. To describe the temporal and spatial variability of PAHs in more detail, the results were also evaluated after being divided into several grouping schemes.

**TABLE 3**
**Statistical Summary of the PAH Concentrations Measured at Each Individual Station during the Study Period (All Units in ng $m^{-3}$)**

| Order | Station Code | BaA | | | CHRY | | | BbF | | | BkF | | | DahA | | | I123P | | | BaP | | | T-PAH | | |
|---|---|---|---|---|---|---|---|---|---|---|---|---|---|---|---|---|---|---|---|---|---|---|---|---|---|
| | | Mean | SD | N | Mean | SD | N | Mean | SD | N | Mean | SD | N | Mean | SD | N | Mean | SD | N | Mean | SD | N | Mean | SD | N |
| 1 | SL1 | 0.51 | 0.78 | 21 | 1.22 | 1.44 | 21 | 2.49 | 3.17 | 18 | 0.98 | 1.04 | 18 | 0.23 | 0.23 | 20 | 0.49 | 0.61 | 20 | 0.84 | 1.11 | 21 | 6.22 | 6.60 | 21 |
| 2 | SL2 | 0.54 | 1.03 | 20 | 1.12 | 1.48 | 20 | 2.22 | 3.35 | 18 | 0.68 | 0.97 | 19 | 0.23 | 0.31 | 17 | 0.49 | 0.73 | 19 | 0.67 | 0.95 | 20 | 5.64 | 7.36 | 20 |
| 3 | SL3 | 0.62 | 1.12 | 21 | 1.12 | 1.46 | 23 | 1.63 | 2.18 | 23 | 0.64 | 0.95 | 20 | 0.19 | 0.20 | 23 | 0.46 | 0.48 | 22 | 0.64 | 0.89 | 22 | 5.11 | 6.23 | 23 |
| 4 | BS1 | 0.17 | 0.17 | 9 | 0.15 | 0.12 | 9 | 0.28 | 0.38 | 15 | 0.17 | 0.16 | 4 | 0.47 | 0.55 | 6 | 0.07 | 0.08 | 10 | 0.09 | 0.08 | 12 | 0.61 | 0.86 | 20 |
| 5 | BS2 | 0.11 | 0.08 | 9 | 0.87 | 1.51 | 9 | 0.35 | 0.42 | 15 | 0.38 | 0.40 | 4 | 0.24 | 0.23 | 7 | 0.15 | 0.22 | 9 | 0.10 | 0.12 | 12 | 0.94 | 1.72 | 21 |
| 6 | DG1 | 0.79 | 0.82 | 16 | 1.10 | 0.98 | 21 | 1.45 | 1.50 | 17 | 0.64 | 0.77 | 14 | 1.75 | 2.14 | 8 | 0.71 | 1.07 | 12 | 0.90 | 1.20 | 14 | 4.75 | 4.02 | 22 |
| 7 | DG2 | 0.65 | 0.66 | 15 | 0.89 | 0.80 | 19 | 1.36 | 1.43 | 17 | 0.38 | 0.37 | 14 | 2.45 | 2.46 | 9 | 0.66 | 0.87 | 16 | 0.63 | 0.62 | 15 | 4.41 | 3.27 | 22 |
| 8 | IC1 | 0.68 | 1.15 | 21 | 1.27 | 1.60 | 22 | 2.34 | 3.04 | 21 | 0.76 | 1.08 | 21 | 0.25 | 0.23 | 16 | 0.52 | 0.63 | 21 | 0.71 | 0.94 | 22 | 6.27 | 7.39 | 23 |
| 9 | IC2 | 0.64 | 1.01 | 21 | 1.12 | 1.39 | 23 | 1.86 | 2.16 | 21 | 0.69 | 0.79 | 22 | 0.24 | 0.20 | 20 | 0.43 | 0.48 | 22 | 0.64 | 0.78 | 23 | 5.33 | 5.71 | 23 |
| 10 | IC3 | 0.31 | 0.38 | 21 | 0.86 | 0.89 | 20 | 1.54 | 2.53 | 20 | 0.54 | 0.70 | 21 | 0.20 | 0.28 | 22 | 0.28 | 0.40 | 22 | 0.49 | 0.57 | 21 | 3.78 | 4.76 | 23 |
| 11 | DJ1 | 0.19 | 0.22 | 15 | 0.37 | 0.64 | 11 | 0.47 | 0.96 | 10 | 1.17 | 1.48 | 6 | 0.19 | 0.17 | 7 | 0.58 | 0.38 | 3 | 0.10 | 0.16 | 11 | 1.27 | 2.71 | 18 |
| 12 | GJ1 | 0.94 | 1.17 | 12 | 0.66 | 0.96 | 14 | 0.62 | 0.59 | 18 | 0.15 | 0.19 | 17 | 0.34 | 0.44 | 13 | 0.27 | 0.29 | 7 | 0.56 | 1.13 | 15 | 2.23 | 2.72 | 22 |
| 13 | GJ2 | 0.76 | 1.20 | 14 | 0.75 | 1.10 | 18 | 0.74 | 0.99 | 21 | 0.35 | 0.70 | 17 | 0.22 | 0.26 | 13 | 0.26 | 0.31 | 8 | 0.78 | 1.47 | 14 | 2.68 | 3.38 | 23 |
| 14 | US1 | 0.45 | 0.64 | 9 | 0.48 | 0.60 | 9 | 0.79 | 1.25 | 12 | 0.24 | 0.11 | 4 | 0.43 | 0.39 | 5 | 0.11 | 0.16 | 8 | 0.08 | 0.11 | 13 | 1.27 | 2.10 | 18 |
| 15 | US2 | 0.26 | 0.36 | 9 | 0.41 | 0.56 | 11 | 0.48 | 0.50 | 13 | 0.18 | 0.08 | 4 | 0.32 | 0.53 | 7 | 0.12 | 0.14 | 9 | 0.16 | 0.22 | 14 | 0.92 | 1.32 | 21 |
| 16 | GW1 | 0.40 | 0.59 | 23 | 0.69 | 0.92 | 23 | 0.81 | 1.19 | 22 | 0.38 | 0.45 | 21 | 0.17 | 0.19 | 15 | 0.21 | 0.25 | 17 | 0.43 | 0.55 | 22 | 2.89 | 3.86 | 23 |
| 17 | GW2 | 0.17 | 0.24 | 22 | 0.33 | 0.38 | 22 | 0.38 | 0.38 | 22 | 0.23 | 0.35 | 20 | 0.12 | 0.10 | 14 | 0.13 | 0.15 | 15 | 0.21 | 0.25 | 20 | 1.38 | 1.68 | 23 |
| 18 | GG1 | 0.66 | 0.82 | 23 | 1.73 | 1.88 | 23 | 2.93 | 3.72 | 21 | 0.92 | 1.12 | 22 | 0.32 | 0.37 | 20 | 0.64 | 0.81 | 21 | 0.95 | 1.08 | 23 | 7.75 | 8.22 | 23 |
| 19 | GG2 | 0.42 | 0.49 | 18 | 1.01 | 1.08 | 20 | 2.67 | 2.95 | 15 | 0.67 | 0.54 | 15 | 0.25 | 0.22 | 17 | 0.52 | 0.61 | 16 | 0.82 | 0.92 | 19 | 5.30 | 5.43 | 20 |
| 20 | GG3 | 1.36 | 1.00 | 3 | 2.51 | 1.84 | 3 | 1.82 | 1.32 | 3 | 1.60 | 1.37 | 3 | 0.22 | 0.18 | 3 | 0.59 | 0.39 | 3 | 1.26 | 0.84 | 3 | 9.36 | 6.85 | 3 |
| 21 | GN1 | 0.08 | 0.14 | 8 | 0.14 | 0.19 | 8 | 0.22 | 0.43 | 13 | 0.46 | 0.66 | 5 | 0.37 | 0.34 | 4 | 0.04 | 0.06 | 8 | 0.05 | 0.09 | 10 | 0.54 | 0.88 | 17 |
| 22 | GN2 | 0.09 | 0.10 | 7 | 0.19 | 0.20 | 11 | 0.35 | 0.56 | 15 | 0.62 | 0.98 | 5 | 0.58 | 0.56 | 4 | 0.03 | 0.05 | 12 | 0.07 | 0.13 | 14 | 0.67 | 1.19 | 22 |
| 23 | GB1 | 0.64 | 0.58 | 15 | 1.37 | 1.14 | 21 | 2.22 | 2.16 | 21 | 0.93 | 1.20 | 17 | 1.54 | 2.12 | 8 | 1.32 | 1.62 | 17 | 0.57 | 0.56 | 15 | 6.26 | 4.04 | 23 |
| 24 | JN1 | 0.86 | 1.18 | 14 | 0.62 | 0.86 | 20 | 0.49 | 0.76 | 20 | 0.17 | 0.20 | 15 | 0.16 | 0.19 | 13 | 0.37 | 0.59 | 6 | 0.38 | 0.85 | 15 | 2.03 | 2.06 | 23 |
| 25 | JN2 | 0.77 | 1.27 | 15 | 0.80 | 1.13 | 16 | 0.69 | 0.87 | 16 | 0.16 | 0.18 | 15 | 0.09 | 0.15 | 11 | 0.22 | 0.23 | 8 | 0.49 | 0.98 | 13 | 2.04 | 2.33 | 23 |
| 26 | JB1 | 0.77 | 1.08 | 14 | 0.67 | 1.46 | 12 | 1.22 | 1.82 | 20 | 0.91 | 1.79 | 11 | 0.21 | 0.27 | 8 | 0.67 | 0.78 | 12 | 1.30 | 1.65 | 9 | 3.25 | 5.88 | 23 |
| 27 | JB2 | 0.78 | 1.07 | 14 | 0.71 | 1.20 | 12 | 1.11 | 1.76 | 15 | 0.78 | 1.62 | 10 | 0.14 | 0.22 | 7 | 0.45 | 0.61 | 11 | 1.29 | 1.66 | 6 | 2.88 | 4.96 | 20 |
| 28 | JB3 | 0.94 | 0.82 | 10 | 0.58 | 0.77 | 12 | 1.16 | 1.53 | 18 | 0.78 | 1.31 | 10 | 0.17 | 0.29 | 11 | 0.34 | 0.36 | 13 | 0.45 | 0.47 | 7 | 2.48 | 3.08 | 22 |
| 29 | CN1 | 0.33 | 0.32 | 16 | 0.36 | 0.57 | 14 | 0.47 | 0.84 | 15 | 0.90 | 1.24 | 8 | 0.14 | 0.16 | 5 | 0.27 | 0.35 | 11 | 0.23 | 0.40 | 9 | 1.60 | 2.99 | 19 |
| 30 | CN2 | 0.39 | 0.27 | 13 | 0.52 | 0.56 | 10 | 0.45 | 0.61 | 15 | 1.06 | 0.77 | 8 | 0.11 | 0.13 | 7 | 0.29 | 0.25 | 8 | 0.16 | 0.23 | 9 | 1.67 | 2.13 | 18 |
| 31 | CN3 | 0.28 | 0.24 | 11 | 0.38 | 0.50 | 12 | 0.57 | 0.88 | 13 | 1.06 | 1.35 | 10 | 0.12 | 0.12 | 7 | 0.25 | 0.30 | 8 | 0.21 | 0.33 | 10 | 1.80 | 2.99 | 17 |
| 32 | CB1 | 0.24 | 0.23 | 17 | 0.28 | 0.43 | 16 | 0.46 | 0.63 | 14 | 1.20 | 1.23 | 11 | 0.15 | 0.14 | 7 | 0.29 | 0.42 | 9 | 0.17 | 0.25 | 11 | 1.77 | 2.39 | 19 |

## RESULTS AND DISCUSSION

### The Fundamental Picture of PAH Distribution

In this study, the concentrations of seven atmospheric PAHs were measured from seven cities and eight provinces over a 2-year time span (February 2006 to January 2008). A statistical summary of all PAH species measured at each individual station is presented in Table 3. To facilitate the comparison of PAH behavior, the concentrations of seven individual PAH species shown in Table 1 were summed up and named the total PAH (T-PAH). According to a comparison of the mean PAH values (Table 3), the peak concentrations of PAH were seen from CHRY and/or BbF compounds, while the lowest values were dominantly seen from DahA. If the T-PAH concentrations are compared among 32 stations, the highest values (ng $m^{-3}$) are typically seen to occur at three stations in GG province (7.75 [GG1], 5.30 [GG2], and 9.36 [GG3]). In contrast, the lowest T-PAH values (ng $m^{-3}$) were recorded in GN province (0.54 [GN1] and 0.67 [GN2]) and BS city (0.61 [BS1] and 0.94 [BS2])

In order to evaluate the extent of the PAH pollution on a wide spatial scale, the concentrations of PAH were evaluated in terms of their values representing city or province scales as shown in Table 4. The plot of the mean T-PAH concentrations between different districts tends to vary greatly on a large spatial scale (Fig. 2). In order to arbitrarily group T-PAH values, a criterion of 4.0 ng $m^{-3}$ was selected to distinguish between high and low PAH data groups based on our PAH distribution patterns. According to this criterion, the high group includes large metropolitan cities (SL, DG, and IC) and two provinces, such as GG and GB. As a result, all the remaining 10 districts can be assorted into the low PAH group, which may represent relatively clean environments with moderately reduced source strengths for PAHs (DJ, GJ, US, GW, GN, JN, JB, CN, CB, and BS). The T-PAH levels seen in US city are unusually low, although the city is well known as an industrial city with huge automobile production facilities. It is thus suspected that the industrial sampling site in US city may not tightly reflect the signature of PAH emissions, unless most industrial sources in US city are effectively releasing PAHs. The results indicated that enhanced BbF values occurred in most cities/provinces in the high PAH group (except for DG city with the highest value of DahA). The relative magnitude of individual PAH compounds was not significantly different in cities/provinces of the low PAH group. However, the least concentrations were commonly seen from either the BaP or DahA compounds in both groups. An investigation of individual PAH compounds in both particle and gaseous phase at Petroleumkaai, Flanders, Belgium (September to October 2001) showed that the mean BbF was 0.21 ng $m^{-3}$, while that of BaP was 0.06 ng $m^{-3}$[4]. Similar to our findings with the highest value of PAH compounds in BbF compound, Guo et al.[9] noticed that exceedingly high concentrations of the BbF compound (10.9 and 5.2 ng $m^{-3}$) occurred at Hunghom and Kwun Tong, Hong Kong, respectively. These authors suggested that BbF was emitted mainly from gasoline-powered vehicular activities, which were the main sources at these areas.

Information regarding the spatial distribution of T-PAHs can provide general views on PAH pollution between different districts. The mean T-PAH levels for the high group were in the range of 4.58 to 6.79 ng $m^{-3}$. These high T-PAH levels in three capital regions (SL, IC, and GG) and two eastern regions (DG and GB) suggest that their source processes may be partially affected by geographic factors. It is reasonable to expect that strong manmade activities in all three districts in and around the capital areas of SL, IC, and GG might have contributed to the considerably strong emissions of PAHs. Similarly, GB province, with the second largest T-PAH levels, consists of a single station representing the Pohang industrial area with the largest steel production facility in Korea. The occurrences of high T-PAH concentrations in GG or IC, however, suggest that the effect of spatial factors on PAH distributions can be highly complicated. Although IC city is the fourth largest city in Korea (2.5 million inhabitants with a harbor, a steel works, and other various industrial activities), the city has three monitoring stations that cover background, residential, and commercial areas[10]. Likewise, although GG province showed the highest T-PAH, the GG3 station in the largest industrial facility is not necessarily recording the highest PAH value of all.

**TABLE 4**
**Concentrations of PAH Measured from Cities (1-7)/Provinces (8-15) during the Study Period (All Units in ng $m^{-3}$)**

| Order | City | Location Code | BaA | CHRY | BbF | BkF | DahA | I123P | BaP | T–PAH |
|---|---|---|---|---|---|---|---|---|---|---|
| 1 | Seoul | SL | 0.56 ± 0.93 (0.24)[1] | 1.09 ± 1.40 (0.56) | 1.80 ± 2.62 (0.73) | 0.71 ± 0.88 (0.33) | 0.19 ± 0.21 (0.09) | 0.44 ± 0.54 (0.18) | 0.70 ± 0.88 (0.39) | 5.31 ± 6.30 (2.71) |
| | | | 0.026 – 4.12 (22)[2] | 0.089 – 5.88 (23) | 0.064 – 10.6 (23) | 0.026 – 3.56 (21) | 0.032 – 0.69 (23) | 0.043 – 2.10 (23) | 0.053 – 3.72 (23) | 0.35 – 22.4 (23) |
| 2 | Busan | BS | 0.13 ± 0.11 (0.11) | 0.39 ± 0.68 (0.09) | 0.31 ± 0.32 (0.18) | 0.27 ± 0.21 (0.21) | 0.35 ± 0.31 (0.26) | 0.10 ± 0.11 (0.06) | 0.10 ± 0.11 (0.06) | 0.73 ± 1.12 (0.20) |
| | | | 0.020 – 0.37 (11) | 0.014 – 2.30 (12) | 0.016 – 1.03 (15) | 0.109 – 0.56 (4) | 0.012 – 0.84 (7) | 0.012 – 0.33 (10) | 0.003 – 0.40 (16) | 0.003 – 4.01 (22) |
| 3 | Daegu | DG | 0.81 ± 0.81 (0.37) | 1.07 ± 0.88 (0.69) | 1.56 ± 1.53 (0.74) | 0.50 ± 0.54 (0.27) | 2.47 ± 2.38 (1.56) | 0.70 ± 0.94 (0.36) | 0.74 ± 0.85 (0.37) | 4.58 ± 3.38 (3.37) |
| | | | 0.064 – 2.66 (17) | 0.120 – 2.77 (21) | 0.158 – 5.18 (19) | 0.063 – 2.06 (15) | 0.228 – 5.84 (10) | 0.059 – 3.21 (16) | 0.125 – 3.18 (15) | 0.89 – 14.0 (22) |
| 4 | Incheon | IC | 0.56 ± 0.77 (0.23) | 1.10 ± 1.24 (0.52) | 1.89 ± 2.41 (1.18) | 0.66 ± 0.79 (0.36) | 0.22 ± 0.20 (0.13) | 0.40 ± 0.48 (0.22) | 0.62 ± 0.68 (0.31) | 5.25 ± 5.66 (3.37) |
| | | | 0.044 – 2.88 (22) | 0.060 – 4.78 (23) | 0.118 – 8.83 (22) | 0.015 – 3.39 (23) | 0.021 – 0.83 (22) | 0.032 – 1.69 (23) | 0.044 – 2.51 (23) | 0.36 – 17.5 (22) |
| 5 | Daejeon | DJ | 0.19 ± 0.22 (0.19) | 0.37 ± 0.64 (0.07) | 0.47 ± 0.96 (0.18) | 1.17 ± 1.48 (0.40) | 0.19 ± 0.17 (0.19) | 0.58 ± 0.38 (0.60) | 0.1 0± 0.16 (0.06) | 1.27 ± 2.71 (0.41) |
| | | | 0.003 – 0.84 (15) | 0.003 – 1.97 (11) | 0.004 – 3.16 (10) | 0.053 – 3.60 (6) | 0.002 – 0.10 (15) | 0.188 – 0.96 (3) | 0.019 – 0.58 (11) | 0.01 – 10.9 (18) |
| 6 | Gwangju | GJ | 0.79 ± 1.10 (0.45) | 0.77 ± 1.07 (0.21) | 0.68 ± 0.77 (0.48) | 0.24 ± 0.37 (0.09) | 0.26 ± 0.32 (0.14) | 0.26 ± 0.22 (0.22) | 0.68 ± 1.11 (0.14) | 2.51 ± 2.73 (1.74) |
| | | | 0.007 – 4.27 (16) | 0.003 – 3.40 (20) | 0.001 – 3.20 (21) | 0.001 – 1.52 (19) | 0.002 – 1.10 (15) | 0.015 – 0.63 (8) | 0.001 – 3.78 (16) | 0.004 – 11.6 (23) |
| 7 | Ulsan | US | 0.33 ± 0.43 (0.14) | 0.38 ± 0.54 (0.19) | 0.61 ± 0.79 (0.30) | 0.21 ± 0.04 (0.20) | 0.32 ± 0.42 (0.06) | 0.11 ± 0.14 (0.05) | 0.11 ± 0.15 (0.07) | 1.01 ± 1.63 (0.20) |
| | | | 0.014 – 1.22 (10) | 0.014 – 1.97 (12) | 0.034 – 2.54 (13) | 0.172 – 0.26 (4) | 0.030 – 1.04 (7) | 0.010 – 0.46 (9) | 0.003 – 0.62 (16) | 0.02– 6.20 (21) |
| 8 | Gangwon | GW | 0.29 ± 0.39 (0.11) | 0.51 ± 0.61 (0.23) | 0.59 ± 0.73 (0.29) | 0.30 ± 0.37 (0.16) | 0.14 ± 0.13 (0.10) | 0.16 ± 0.19 (0.08) | 0.30 ± 0.37 (0.14) | 2.14 ± 2.60 (1.10) |
| | | | 0.008 – 1.44 (23) | 0.043 – 2.27 (23) | 0.041 – 2.76 (22) | 0.029 – 1.63 (21) | 0.007 – 0.40 (16) | 0.002 – 0.63 (18) | 0.008 – 1.44 (23) | 0.12 – 9.24 (23) |
| 9 | Gyunggi | GG | 0.59 ± 0.69 (0.30) | 1.47 ± 1.55 (0.88) | 2.47 ± 3.12 (1.44) | 0.81 ± 0.89 (0.51) | 0.29 ± 0.27 (0.24) | 0.54 ± 0.66 (0.33) | 0.89 ± 0.83 (0.63) | 6.79 ± 6.83 (4.70) |
| | | | 0.009 – 2.51 (23) | 0.049 – 6.11 (23) | 0.072 – 11.8 (22) | 0.094 – 3.34 (22) | 0.031 – 1.16 (21) | 0.026 – 3.02 (22) | 0.066 – 3.14 (23) | 0.34 – 24.4 (23) |
| 10 | Gyungnam | GN | 0.07 ± 0.08 (0.05) | 0.16 ± 0.18 (0.15) | 0.30 ± 0.48 (0.16) | 0.54 ± 0.82 (0.08) | 0.48 ± 0.33 (0.56) | 0.03 ± 0.04 (0.02) | 0.05 ± 0.10 (0.01) | 0.56 ± 0.97 (0.12) |
| | | | 0.006 – 0.24 (9) | 0.010 – 0.63 (12) | 0.017 – 1.96 (15) | 0.057 – 1.96 (5) | 0.018 – 0.77 (4) | 0.003 – 0.12 (12) | 0.001 – 0.38 (14) | 0.003 – 4.25 (22) |
| 11 | Gyungbuk | GB | 0.64 ± 0.58 (0.43) | 1.37 ± 1.14 (0.81) | 2.22 ± 2.16 (1.40) | 0.99 ± 1.22 (0.48) | 1.54 ± 2.12 (0.45) | 0.32 ± 1.62 (0.35) | 0.57 ± 0.56 (0.36) | 6.26 ± 4.04 (5.32) |
| | | | 0.221 – 2.52 (15) | 0.066 – 4.63 (21) | 0.339 – 6.86 (21) | 0.025 – 3.39 (16) | 0.163 – 5.05 (8) | 0.078 – 5.27 (17) | 0.033 – 2.00 (15) | 1.56 – 16.6 (23) |
| 12 | Junnam | JN | 0.84 ± 1.16 (0.24) | 0.64 ± 0.89 (0.30) | 0.52 ± 0.74 (0.19) | 0.15 ± 0.17 (0.09) | 0.13 ± 0.13 (0.08) | 0.25 ± 0.32 (0.08) | 0.39 ± 0.85 (0.08) | 2.03 ± 2.05 (1.57) |
| | | | 0.002 – 3.89 (16) | 0.004 – 3.48 (20) | 0.004 – 2.71 (21) | 0.001 – 0.70 (17) | 0.002 – 0.42 (15) | 0.006 – 0.89 (8) | 0.004 – 3.47 (16) | 0.01 – 7.50 (23) |
| 13 | Jeonbuk | JB | 0.81 ± 0.81 (0.56) | 0.51 ± 0.89 (0.31) | 1.10 ± 1.58 (0.28) | 0.68 ± 1.37 (0.22) | 0.12 ± 0.17 (0.04) | 0.46 ± 0.51 (0.21) | 0.92 ± 1.01 (0.47) | 2.83 ± 4.40 (1.45) |
| | | | 0.032 – 2.95 (18) | 0.004 – 3.89 (18) | 0.020 – 5.21 (21) | 0.001 – 5.21 (14) | 0.006 – 0.49 (14) | 0.011 – 1.84 (16) | 0.051 – 2.73 (11) | 0.04 – 21.1 (23) |
| 14 | Chungnam | CN | 0.29 ± 0.22 (0.22) | 0.31 ± 0.47 (0.06) | 0.41 ± 0.68 (0.16) | 0.86 ± 1.09 (0.36) | 0.09 ± 0.11 (0.05) | 0.22 ± 0.25 (0.08) | 0.16 ± 0.27 (0.02) | 1.63 ± 2.53 (0.71) |
| | | | 0.017 – 0.76 (19) | 0.006 – 1.51 (17) | 0.016 – 2.48 (19) | 0.027 – 3.45 (16) | 0.002 – 0.30 (10) | 0.002 – 0.76 (14) | 0.004 – 0.84 (14) | 0.05 – 9.48 (19) |
| 15 | Chungbuk | CB | 0.24 ± 0.23 (0.16) | 0.28 ± 0.43 (0.06) | 0.46 ± 0.63 (0.33) | 1.20 ± 1.23 (0.88) | 0.15 ± 0.14 (0.07) | 0.29 ± 0.42 (0.13) | 0.17 ± 0.25 (0.08) | 1.77 ± 2.39 (0.73) |
| | | | 0.011 – 0.76 (17) | 0.001 – 1.56 (16) | 0.001 – 2.40 (14) | 0.036 – 3.22 (11) | 0.024 – 0.37 (7) | 0.004 – 1.22 (9) | 0.004 – 0.86 (11) | 0.04 – 9.38 (19) |

[1] Mean ± SD (median)

[2] Min-Max (number).

## The Distribution of PAH between Different Land Use Types

To estimate the effects of manmade activities on the distribution of PAHs, the concentration levels of PAHs are compared among land use criteria such as background (B), greenland (G), residential (R), commercial (C), and industrial (I) (Table 2). To this end, each individual station was sorted out according to land use criteria (Fig. 3A). According to these criteria, the distribution of PAHs appears to be distinguishable from each other. A comparison of T-PAH levels among different land use types indicated that the enhanced T-PAH concentration levels were observed most frequently from C as 4.85 ng $m^{-3}$, due probably to effects of transportation sources. This was then followed by R and I as 4.42 and 4.27 ng $m^{-3}$, respectively. In contrast, the lowest values of T-PAH occurred from G (3.09 ng $m^{-3}$) and B type (2.60 ng $m^{-3}$). The relative patterns derived by this classification are highly consistent in terms of the magnitude of individual PAHs or T-PAHs. The highest values of some PAHs (e.g., CHRY, BbF, and BaP) were seen most frequently at C type, while I type was dominated by BaA and BkF. In contrast, DahA recorded the highest value from land used type of R. It was noted that most PAH compounds consistently showed the lowest values at either G or B type.

The criteria of land use types were also evaluated in relative terms for all PAH data (through a normalization) (Fig. 3B). The extent of the T-PAH pollution was found in the order of C > R > I > G > B. The prominence of T-PAHs in commercial and industrial areas has been reported in many previous studies. For example, based on the investigation of total seven PAH concentration in Houston, Texas, Fraser et al.[11] suggested that the PAH levels in the industrial area of Clinton (7.2 ng $m^{-3}$ during spring 1997) were significantly higher than the background area of Galveston (0.09 ng $m^{-3}$ during March 1997 to

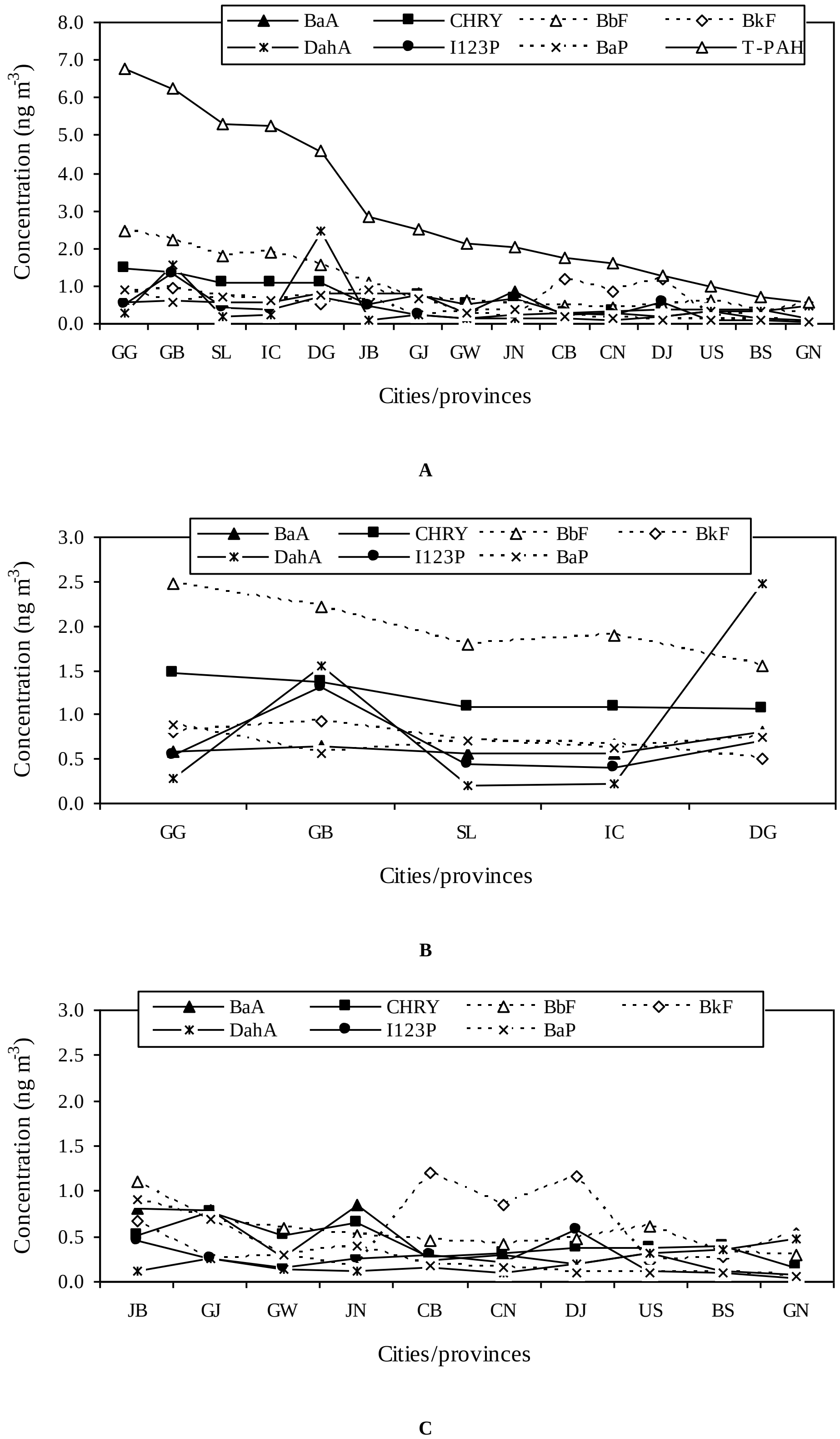

**FIGURE 2**. Spatial distribution of the PAH concentrations among seven cities and eight provinces in Korea (2006–2008). (A) Comparison of T-PAH and individual species for all cities/provinces; (B) comparison of individual PAH species for high concentration groups; (C) comparison of individual PAH species for low concentration groups.

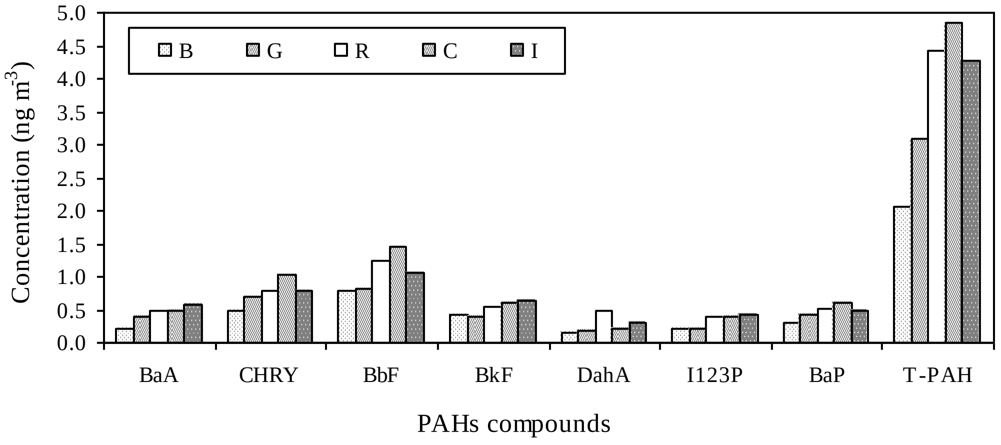

**A**

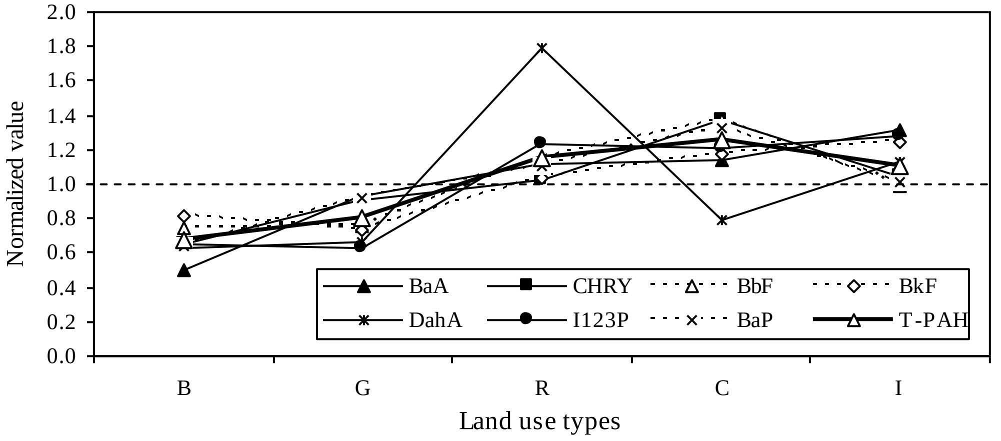

**B**

**FIGURE 3.** Comparison of the mean PAH concentration levels between land use types. (A) Absolute PAH concentration levels for five land use types; (B) normalization of PAH concentration levels.

March 1998). Similarly, a study conducted in Flanders, Belgium (2001–2002) showed that T-PAH levels for Zelzate (steel industry site), Borgerhout (urban area), and Wingene (rural area) were 8.73, 4.04, and 0.59 ng $m^{-3}$, respectively[4]. However, highly contrasting patterns were also observed from three sites in Lahore, Pakistan (1992–1993) in which the highest T-PAH concentration occurred at a rural site (57.2) followed by a city (53.9) and industrial site (45.3 ng $m^{-3}$)[12]. The presence of unusually high PAH levels at that rural site was ascribed to the dozen or so brick kilns that are approximately 3 km apart (coal, furnace oil, sawdust, and rubber tires), while cooking fuels at the rural site can also release PAHs.

The relative enhancement of the T-PAHs in commercial areas can be explained by dense population and heavy traffic, as fossil fuel combustion for heating and traffic exhaust can promote enhanced emissions of PAHs. Muller et al.[13] reported that the higher the number of vehicles (especially of heavy vehicles) at a site, the higher the total concentration of PAHs there. In addition, Smith et al.[12] suggested that vehicular activity was one of the major sources of PAHs, especially ones with the higher molecular weight in the city and industrial sites. The major contributors of this vehicular emission are often designated as two-stroke-engine-powered motorcycles (which add oil to the petrol to compensate for the lack of a wet sump), four-cylinder petrol vehicles running on leaded fuel, and old diesel-powered heavy vehicles[12]. Moreover, if the general road conditions were poor with insufficient land markings, poor surface and drainage conditions can also contribute to high PAH emissions[12].

## Temporal Variation Patterns of PAHs in the Study Area

To examine the temporal variabilities of PAHs, all of our data were examined at both monthly and seasonal intervals. As shown in Fig. 4, the monthly variation patterns of T-PAHs (Fig. 4) indicate a strong seasonality with a minimum in July (1.25 ng $m^{-3}$) and a maximum in December (7.49 ng $m^{-3}$). As such, comparisons based on the seasonal mean PAH values consistently indicate the peak occurrences in winter, with an exception of DahA (Fig. 5); it exhibits a maximum in fall (0.91 ng $m^{-3}$). The importance of meteorological parameters (e.g., temperature, humidity, precipitation, etc.) is seen as critical in controlling the environmental distribution of PAHs. Li et al.[14] demonstrated that the typical subtropical monsoon climate in south China, with cool and dry in winter and hot and humid in summer, may play a key role in controlling the source seasonality of PAHs (by enhancing vehicle exhaust in winter and ground evaporation in summer). As rainfall occurs most intensively in the summer months, it can effectively wash out PAHs (T-PAHs in summer: 1.42 ng $m^{-3}$). In addition, photochemical degradation of PAHs can also occur under high solar radiation in summer seasons[15]. The T-PAH concentrations then began to increase gradually from fall (2.37 ng $m^{-3}$) through winter (5.60 ng $m^{-3}$), with an increase in anthropogenic emissions (e.g., household heating). In fact, this wintertime enhancement in PAH levels is also favored by such factors as (a) suppression of vertical dispersion (due to inversion and lower mixing layer), (b) less-intensive atmospheric reactions, and (c) enhanced sorption to particles at low temperature (as a result of reduced vapor pressure and/or shifting in the gas/particle distribution at low temperature)[6].

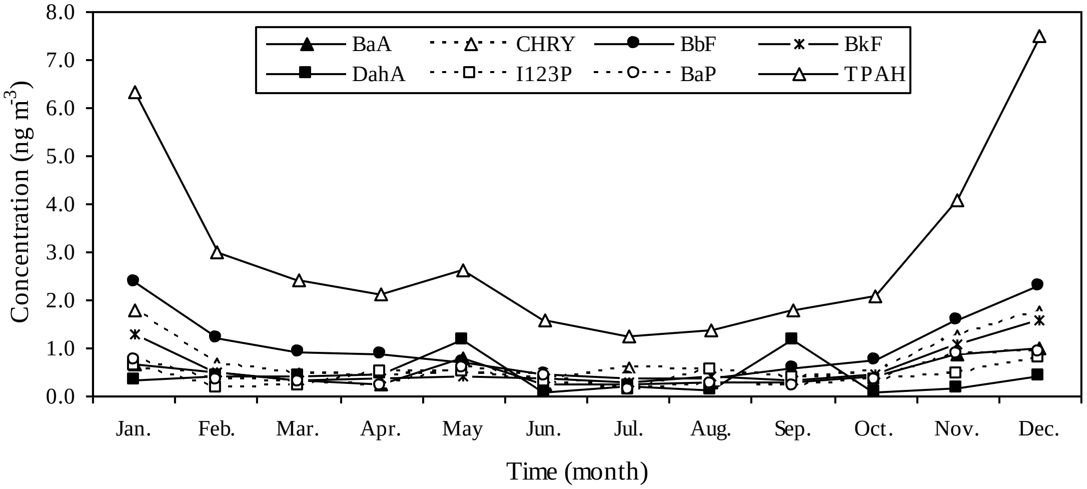

**FIGURE 4.** The monthly variation of the PAH concentrations for all seven cities and eight provinces during the entire study period.

As a means to examine the interannual changes in PAH distribution, differences in the concentration levels between the 2-year period (2006 and 2007) were also compared (Table 5). When the annual mean concentration levels of T-PAHs were compared between 2006 and 2007, 12 out of all 15 cities/provinces showed a relative increase from 2006 to 2007. Increases in T-PAH concentration were seen from large-scale cities like SL (from 4.61 [2006] to 5.66 ng $m^{-3}$ [2007]) and those surrounded by large suburban and rural areas such as JB (1.69 to 4.18 ng $m^{-3}$) and GJ (1.46 to 3.62 ng $m^{-3}$). According to the results of a t-test, the difference in T-PAH concentration levels during the 2-year period was not statistically significant in most

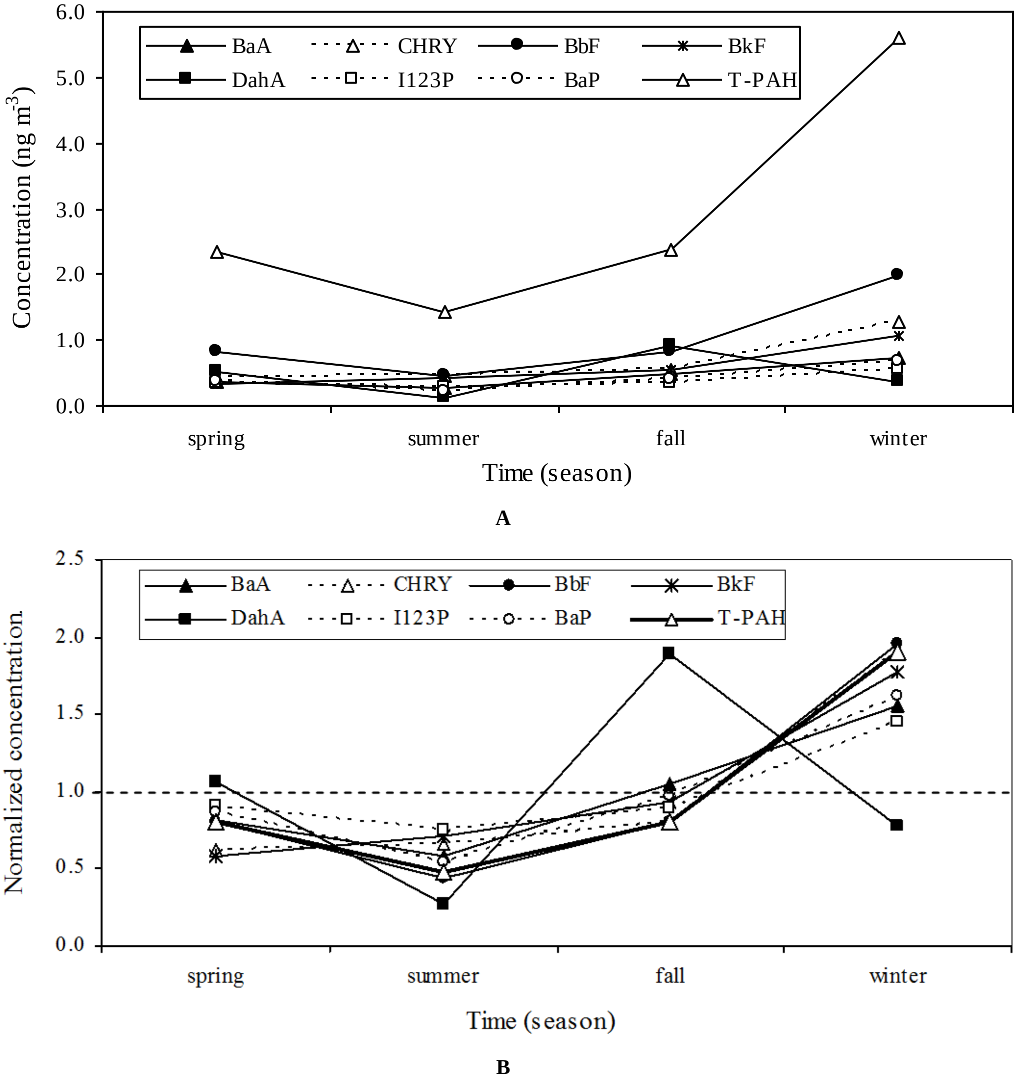

**FIGURE 5.** Seasonal patterns of the PAH concentration during study period. (A) Seasonal mean values; (B) normalization of seasonal mean values.

districts except for GB province. However, GB province experienced the strongest reduction in T-PAH levels, with 9.37 (2006) to 3.27 ng $m^{-3}$ (2007). This comparison thus suggests that the distribution of PAHs is yet not simple enough to build an interannual trend in most cases.

## Factors Affecting PAH Distributions among Different Sites

To assess the major factors affecting the emission levels of PAHs, source characteristics of different PAHs need to be examined accurately. Chang et al.[16] listed the indicatory PAHs for various major sources: (1) CHRY is emitted from incineration, diesel and gasoline vehicle emission, and industrial oil burning; (2) BaP is emitted from the steel industry, industrial oil burning, and indoor sources (e.g., wood burning, incense burning, and cooking fuel); (3) I123D is typically used as a marker for gasoline vehicles, incineration, and incomplete combustion and pyrolysis of fuels; (4) power plants are a major source of the DahA compound; and (5) the steel industry is often designated as the most important source of BaA[16].

**TABLE 5**
**Results of t-Test for PAH Concentrations of a Given City Measured between 2006 and 2007**

| | SL | BS | DG | IC | DJ | GJ | US | GW | GG | GN | GB | JN | JB | CN | CB |
|---|---|---|---|---|---|---|---|---|---|---|---|---|---|---|---|
| *The mean ± SD values of the individual PAH compounds in 2006* | | | | | | | | | | | | | | | |
| BaA | 0.43 ± 0.60 | 0.06 ± 0.08 | 1.61 ± 0.90 | 0.29 ± 0.25 | 0.16 ± 0.12 | 0.47 ± 0.80 | 0.04 ± 0.02 | 0.24 ± 0.32 | 0.49 ± 0.58 | 0.02 ± 0.01 | 1.13 ± 1.21 | 0.47 ± 1.01 | 0.63 ± 0.35 | 0.23 ± 0.21 | 0.21 ± 0.29 |
| CHRY | 0.78 ± 0.75 | 0.04 ± 0.02 | 1.57 ± 0.86 | 0.76 ± 0.69 | 0.16 ± 0.26 | 0.27 ± 0.52 | 0.04 ± 0.03 | 0.45 ± 0.59 | 1.19 ± 1.03 | 0.04 ± 0.06 | 2.19 ± 1.18 | 0.47 ± 0.67 | 0.38 ± 0.30 | 0.04 ± 0.02 | 0.03 ± 0.03 |
| BbF | 1.97 ± 2.34 | 0.14 ± 0.16 | 2.80 ± 1.69 | 2.24 ± 2.38 | 0.54 | 0.35 ± 0.27 | 0.18 ± 0.13 | 0.66 ± 0.82 | 3.34 ± 3.42 | 0.15 ± 0.16 | 4.20 ±1.92 | 0.17 ± 0.19 | 0.34 ± 0.60 | 0.13 ± 0.11 | 0.02 ± 0.02 |
| BkF | 0.49 ± 0.52 | | 0.85 ± 1.09 | 0.44 ± 0.45 | | 0.22 ± 0.44 | | 0.21 ± 0.21 | 0.62 ± 0.51 | | 2.14 ± 1.49 | 0.09 ± 0.07 | 0.22 ± 0.24 | 0.37 ± 0.36 | 0.39 ± 0.58 |
| DahA | 0.21 ± 0.20 | 0.02 ± 0.01 | 3.86 ± 2.09 | 0.25 ± 0.25 | 0.24 ± 0.20 | 0.22 ± 0.33 | 0.05 ± 0.02 | 0.14 ± 0.14 | 0.39 ± 0.32 | | 3.43 ± 2.66 | 0.12 ± 0.14 | 0.02 ± 0.02 | 0.04 ± 0.04 | 0.20 ± 0.24 |
| I123P | 0.35 ± 0.41 | 0.02 ± 0.01 | 1.70 ± 1.20 | 0.35 ± 0.47 | | 0.26 ± 0.22 | 0.02 ± 0.002 | 0.17 ± 0.02 | 0.68 ± 0.89 | 0.01 ± 0.01 | 3.24 ± 1.20 | 0.25 ± 0.32 | 0.38 ± 0.37 | 0.16 ± 0.21 | 0.31 ± 0.61 |
| BaP | 0.62 ± 0.64 | 0.05 ± 0.07 | 1.62 ± 1.53 | 0.52 ± 0.50 | 0.04 ± 0.04 | 0.20 ± 0.26 | 0.03 ± 0.03 | 0.23 ± 0.28 | 0.91 ± 0.74 | 0.01 ± 0.01 | 1.28 ± 0.82 | 0.11 ± 0.12 | 0.98 ± 1.18 | 0.02 ± 0.01 | 0.02 ± 0.01 |
| T-PAH | 4.61 ± 4.34 | 0.15 ± 0.14 | 6.15 ± 3.44 | 4.53 ± 4.37 | 0.55 ± 0.59 | 1.46 ± 1.40 | 0.15 ± 0.11 | 1.97 ± 0.24 | 7.16 ± 6.52 | 0.13 ± 0.17 | 9.37 ± 3.55 | 1.23 ± 1.08 | 1.69 ± 2.12 | 0.62 ± 0.61 | 0.62 ± 1.22 |
| *The mean ± SD values of the individual PAH compounds in 2007* | | | | | | | | | | | | | | | |
| BaA | 0.63 ± 1.24 | 0.19 ± 0.11 | 0.43 ± 0.49 | 0.69 ± 0.96 | 0.14 ± .15 | 1.04 ± 1.27 | 0.37 ± 0.36 | 0.27 ± 0.42 | 0.57 ± 0.73 | 0.08 ± 0.05 | 0.49 ± 0.29 | 1.21 ± 1.25 | 1.01 ± 1.01 | 0.28 ± 0.19 | 0.20 ± 0.13 |
| CHRY | 1.27 ± 1.85 | 0.48 ± 0.81 | 0.66 ± 0.74 | 1.20 ± 1.45 | 0.24 ± 0.45 | 1.26 ± 1.27 | 0.39 ± 0.17 | 0.50 ± 0.65 | 1.61 ± 1.98 | 0.24 ± 0.20 | 0.75 ± 0.65 | 0.82 ± 1.07 | 0.67 ± 1.24 | 0.36 ± 0.42 | 0.30 ± 0.32 |
| BbF | 1.62 ± 3.10 | 0.40 ± 0.33 | 0.80 ± 0.88 | 1.47 ± 2.59 | 0.12 ± 0.11 | 0.99 ± 1.02 | 0.72 ± 0.84 | 0.48 ± 0.67 | 1.73 ± 2.91 | 0.42 ± 0.68 | 0.66 ± 0.33 | 0.94 ± 0.98 | 1.89 ± 1.96 | 0.40 ± 0.58 | 0.40 ± 0.28 |
| BkF | 0.86 ± 1.15 | 0.18 ± 0.11 | 0.43 ± 0.38 | 0.73 ± 0.92 | 0.75 ± 1.12 | 0.27 ± 0.27 | 0.19 ± 0.03 | 0.35 ± 0.47 | 0.92 ± 1.16 | 0.66 ± 0.90 | 0.62 ± 0.91 | 0.22 ± 0.23 | 1.15 ± 2.15 | 1.04 ± 1.26 | 1.32 ± 1.30 |
| DahA | 0.17 ± 0.24 | 0.48 ± 0.27 | 0.44 ± 0.17 | 0.18 ± 0.16 | 0.11 ± 0.17 | 0.34 ± 0.35 | 0.53 ± 0.46 | 0.14 ± 0.14 | 0.17 ± 0.15 | 0.48 ± 0.33 | 0.44 ± 0.25 | 0.16 ± 0.14 | 0.23 ± 0.21 | 0.13 ± 0.14 | 0.08 ± 0.08 |
| I123P | 0.51 ± 0.67 | 0.13 ± 0.09 | 0.25 ± 0.18 | 0.40 ± 0.49 | 0.39 ± 0.29 | | 0.10 ± 0.06 | 0.13 ± 0.19 | 0.39 ± 0.38 | 0.05 ± 0.05 | 0.24 ± 0.17 | | 0.62 ± 0.72 | 0.19 ± 0.21 | 0.16 ± 0.08 |
| BaP | 0.74 ± 1.13 | 0.12 ± 0.13 | 0.51 ± 0.51 | 0.59 ± 0.73 | 0.15 ± 0.21 | 1.48 ± 1.54 | 0.16 ± 0.20 | 0.32 ± 0.42 | 0.82 ± 0.96 | 0.09 ± 0.12 | 0.36 ± 0.32 | 1.00 ± 1.41 | 1.02 ± 0.93 | 0.18 ± 0.24 | 0.14 ± 0.11 |
| T-PAH | 5.66 ± 8.15 | 1.13 ± 1.28 | 3.15 ± 2.94 | 5.11 ± 6.47 | 0.79 ± 1.58 | 3.62 ± 3.44 | 1.36 ± 1.40 | 2.03 ± 2.80 | 6.08 ± 7.62 | 0.98 ± 1.30 | 3.27 ± 1.60 | 2.97 ± 2.48 | 4.19 ± 5.83 | 1.56 ± 2.10 | 1.81 ± 1.63 |
| *Results of t-test between the 2-year data sets (Probability)* | | | | | | | | | | | | | | | |
| BaA | 0.53 | 0.04 | 0.04 | 0.12 | 0.62 | 0.29 | 0.06 | 0.60 | 0.53 | 0.06 | 0.47 | 0.22 | 0.39 | 0.40 | 0.75 |
| CHRY | 0.31 | 0.10 | 0.03 | 0.21 | 0.35 | 0.04 | 0.05 | 0.68 | 0.42 | 0.02 | 0.01 | 0.39 | 0.58 | 0.03 | 0.03 |
| BbF | 0.77 | 0.04 | 0.02 | 0.55 | | 0.05 | 0.07 | 0.66 | 0.25 | 0.28 | 0.0006 | 0.04 | 0.04 | 0.09 | 0.02 |
| BkF | 0.29 | | 0.55 | 0.20 | | 0.76 | | 0.26 | 0.35 | | 0.13 | 0.15 | 0.25 | 0.09 | 0.09 |
| DahA | 0.66 | 0.02 | 0.01 | 0.50 | 0.59 | 0.48 | 0.13 | 0.87 | 0.06 | | 0.19 | 0.56 | 0.04 | 0.17 | 0.75 |
| I123P | 0.44 | 0.03 | 0.05 | 0.65 | | | 0.09 | 0.84 | 0.39 | 0.08 | 0.002 | | 0.53 | 0.45 | 0.94 |
| BaP | 0.67 | 0.12 | 0.34 | 0.50 | 0.25 | 0.10 | 0.07 | 0.38 | 0.94 | 0.11 | 0.20 | 0.23 | 0.83 | 0.07 | 0.06 |
| T-PAH | 0.62 | 0.02 | 0.05 | 0.57 | 0.29 | 0.07 | 0.02 | 0.77 | 0.81 | 0.04 | 0.0002 | 0.07 | 0.24 | 0.10 | 0.06 |

Khalili et al.[17] suggested that diesel-powered vehicles are a source of BkF. As reported by a number of previous studies, BbF was known as a source marker for gasoline emission[9], diesel-powered vehicles[17], and stationary emissions sources[4]. Using a chemical mass balance receptor model, the relative contribution of the various primary sources of PAHs at downtown Los Angeles in 1982 was estimated to predict the major source of BaA from natural gas combustion, most I123D from gasoline vehicles, and a small part of I123D from wood combustion[18]. Similarly, BkF and BbF were also predicted to come from such sources as gasoline vehicles, natural gas combustion, wood combustion, and diesel vehicles[18]. Applying the same model to PAH data collected from Fresno, CA (December, 1995), Schauer and Cass[19] suggested that the main sources of BbF, BkF, and BaA were emitted from natural gas combustion, followed by softwood combustion, hardwood combustion, gasoline vehicles, and meat charbroiling. However, the major sources of I123D at Fresno were allocated to softwood combustion, hardwood combustion, gasoline vehicles, meat charbroiling, and diesel exhaust[19]. In an estimate of source seasonality for PAHs in a subtropical city (Guangzhou, South China), the emissions from vehicular exhaust accounted for 69% of the particulate PAHs, while coal combustion contributed to the remaining 31%[14].

To examine the factors affecting PAH distribution in the study area, the concentration data of all seven PAHs were analyzed by correlation analysis (Table 6). For this purpose, a correlation analysis was conducted between different sites (for each PAH compound: type I) and between different PAH compounds (at a given study site: type II). The strengths of the correlation between different data pairs are then compared arbitrarily by dividing them into five categories of correlation strengths based on the magnitude of probability (P): no class: $r \leq 0.2$; class I: $0.2 < r \leq 0.5$; class II: $0.5 < r \leq 0.8$; class III: $r > 0.8$. The results of correlation analysis type I indicated that the occurrences of strongly correlated cases (class III) were abundant for compounds that are emitted from mobile and industrial sources, such as DahA, BbF, I123P, and BaP with their occurrence frequencies of 58, 55, 51, and 48%, respectively. In contrast, such relationships were found rather scarcely from BaA (41%) and CHRY (32%). The results of correlation analysis type II indicate that the strong correlations were found most frequently in industrial cities and cities with high population density (e.g., SL, BS, IC, and US). On the other hand, most weak

## TABLE 6
## Summary of the Correlation Analysis Made using Individual PAH Data Measured from Seven Cities and Eight Provinces in Korea

### A. Results of the correlation analysis between cities/provinces for each PAH compound

| Classify | PAH Compound | | | | | | | | | | | | | |
|---|---|---|---|---|---|---|---|---|---|---|---|---|---|---|
| | BaA | | CHRY | | BbF | | BkF | | DahA | | I123P | | BaP | |
| No class | 6 | 6% | 15 | 14% | 14 | 13% | 21 | 20% | 7 | 7% | 11 | 10% | 11 | 10% |
| I | 26 | 25% | 23 | 22% | 14 | 13% | 17 | 16% | 20 | 19% | 25 | 24% | 17 | 16% |
| II | 30 | 29% | 33 | 31% | 19 | 18% | 20 | 19% | 17 | 16% | 15 | 14% | 27 | 26% |
| III | 43 | 41% | 34 | 32% | 58 | 55% | 47 | 45% | 61 | 58% | 54 | 51% | 50 | 48% |

### B. Results of the correlations analysis between 7 PAH compounds for each city and/or province

| PAH Compounds | Classify | Cities/Provinces | | | | | | | | | | | | | | |
|---|---|---|---|---|---|---|---|---|---|---|---|---|---|---|---|---|
| | | SL | BS | DG | IC | DJ | GJ | US | GW | GG | GN | GB | JN | JB | CN | CB |
| | No class | 6 | 6 | 8 | 5 | 4 | 8 | 5 | 2 | 6 | 6 | 6 | 7 | 8 | 4 | 7 |
| BaA | I | 5 | 4 | 5 | 3 | 2 | 4 | 2 | 7 | 5 | 3 | 5 | 3 | 5 | 5 | 2 |
| | II | 2 | 4 | 0 | 6 | 6 | 1 | 7 | 5 | 2 | 4 | 2 | 3 | 1 | 4 | 4 |
| | III | 1 | 0 | 1 | 0 | 2 | 1 | 0 | 0 | 1 | 1 | 1 | 1 | 0 | 1 | 1 |
| | No class | 2 | 3 | 5 | 1 | 2 | 9 | 3 | 3 | 3 | 2 | 10 | 8 | 4 | 6 | 7 |
| CHRY | I | 5 | 3 | 8 | 4 | 3 | 3 | 6 | 5 | 6 | 5 | 3 | 4 | 5 | 3 | 4 |
| | II | 4 | 2 | 1 | 5 | 6 | 2 | 4 | 5 | 3 | 5 | 0 | 2 | 4 | 3 | 1 |
| | III | 3 | 6 | 0 | 4 | 3 | 0 | 1 | 1 | 2 | 2 | 1 | 0 | 1 | 2 | 2 |
| | No class | 9 | 5 | 12 | 10 | 3 | 8 | 5 | 8 | 10 | 7 | 11 | 5 | 8 | 6 | 9 |
| BbF | I | 3 | 1 | 1 | 2 | 4 | 1 | 2 | 6 | 2 | 2 | 3 | 7 | 1 | 2 | 1 |
| | II | 0 | 5 | 1 | 2 | 2 | 4 | 2 | 0 | 0 | 3 | 0 | 1 | 3 | 4 | 3 |
| | III | 2 | 3 | 0 | 0 | 5 | 1 | 5 | 0 | 2 | 2 | 0 | 1 | 2 | 2 | 1 |
| | No class | 3 | 8 | 5 | 3 | 5 | 7 | 6 | 3 | 3 | 7 | 13 | 10 | 7 | 4 | 10 |
| BbF | I | 2 | 1 | 5 | 3 | 3 | 4 | 2 | 0 | 5 | 2 | 0 | 2 | 2 | 6 | 3 |
| | II | 5 | 1 | 2 | 2 | 4 | 1 | 1 | 7 | 4 | 2 | 1 | 1 | 2 | 1 | 0 |
| | III | 4 | 4 | 2 | 6 | 2 | 2 | 5 | 4 | 2 | 3 | 0 | 1 | 3 | 3 | 1 |
| | No class | 2 | 6 | 12 | 4 | 11 | 5 | 8 | 11 | 7 | 12 | 12 | 5 | 9 | 7 | 7 |
| DahA | I | 5 | 1 | 2 | 5 | 2 | 4 | 3 | 2 | 3 | 0 | 0 | 4 | 2 | 3 | 0 |
| | II | 7 | 3 | 0 | 3 | 1 | 4 | 1 | 1 | 4 | 1 | 2 | 4 | 2 | 4 | 5 |
| | III | 0 | 4 | 0 | 2 | 0 | 1 | 2 | 0 | 0 | 1 | 0 | 1 | 1 | 0 | 2 |
| | No class | 5 | 2 | 11 | 6 | 5 | 11 | 7 | 8 | 5 | 11 | 11 | 6 | 3 | 3 | 6 |
| I123P | I | 3 | 5 | 2 | 2 | 1 | 0 | 2 | 2 | 3 | 1 | 1 | 6 | 2 | 2 | 1 |
| | II | 5 | 3 | 1 | 2 | 5 | 3 | 4 | 3 | 6 | 2 | 1 | 2 | 6 | 6 | 3 |
| | III | 1 | 4 | 0 | 4 | 3 | 0 | 1 | 1 | 0 | 0 | 1 | 0 | 3 | 3 | 4 |
| | No class | 5 | 8 | 9 | 6 | 4 | 10 | 8 | 4 | 8 | 10 | 7 | 7 | 5 | 4 | 4 |
| BaP | I | 4 | 3 | 4 | 2 | 5 | 2 | 4 | 3 | 4 | 2 | 4 | 4 | 6 | 5 | 4 |
| | II | 3 | 1 | 0 | 4 | 4 | 1 | 0 | 7 | 0 | 0 | 2 | 1 | 2 | 4 | 4 |
| | III | 2 | 2 | 1 | 2 | 1 | 1 | 2 | 0 | 2 | 2 | 1 | 2 | 1 | 1 | 2 |
| | No class | 32[1] (33%)[2] | 38 (39%) | 62 (63%) | 35 (36%) | 34 (35%) | 58 (59%) | 42 (43%) | 39 (40%) | 42 (43%) | 55 (56%) | 70 (71%) | 48 (49%) | 44 (45%) | 34 (35%) | 50 (50%) |
| Total | I | 27 (28%) | 18 (18%) | 27 (28%) | 21 (21%) | 20 (20%) | 18 (18%) | 21 (21%) | 25 (26%) | 28 (29%) | 15 (15%) | 16 (16%) | 30 (31%) | 23 (23%) | 26 (27%) | 15 (15%) |
| | II | 26 (27%) | 19 (19%) | 5 (5%) | 24 (24%) | 28 (29%) | 16 (16%) | 19 (19%) | 28 (29%) | 19 (9%) | 17 (17%) | 8 (8%) | 14 (14%) | 20 (20%) | 26 (27%) | 20 (20%) |
| | III | 13 (13%) | 23 (23%) | 4 (4%) | 18 (18%) | 16 (16%) | 6 (6%) | 16 (16%) | 6 (6%) | 9 (9%) | 11 (11%) | 4 (4%) | 6 (6%) | 11 (11%) | 12 (12%) | 13 (13%) |

1 Number of correlation pairs.

2 Percentage of correlation pairs.

correlation pairs (e.g., no class or class I) occurred abundantly between stations with the low T-PAH concentrations, such as JN, GN, and GJ. It thus appears that spatial factors may greatly contribute to the distribution of PAHs in air, although the differences in their concentration levels are not that distinct relative to land use types.

## Comparison with PAH Concentration Measured in the Previous Studies

To estimate the pollution status of seven PAH compounds across all sites in this study, their concentration levels were compared with those reported previously from various locations around the globe (Table 7). In light of differences in the measurement methods, the PAH concentration data were examined to include three types of data including gas, particle, and the sum of both phases. Based on this criterion, the PAH data were compared between three major land use types: (1) industrial areas directly impacted by industrial source processes, (2) urban areas (commercial and residential), and (3) background areas (suburban, rural, and greenland). In general, the concentration of PAH species with lighter molecular weights (MW) was predominantly higher in the gaseous phase than that of the particle phase, regardless of site types. Ravindra et al.[4] estimated that the concentration of PAHs present in the gaseous phase was more than 80% of the T-PAH concentration. Among the industrial areas, the highest T-PAH concentration (14.5 ng $m^{-3}$ in PM10) was recorded in Kwun Tong in Hong Kong, China during 2000–2001[9]. It was then followed by Taichung (Taiwan) (13.5 ng $m^{-3}$ in TSP) in 2002[20]. Because of great improvements in control technologies, the T-PAH levels in the industrial areas in Europe or North America, however, show significant reductions relative to others. For example, the lowest T-PAH values of 0.95 (in PM2.5) and of 0.96 ng $m^{-3}$ (in sum phase) were measured in the industrial area at Clinton (Houston, Texas) in 1997[11] and at Petroleumkaai (Flanders, Belgium) in the vicinity of the industrial activities (oil refineries and petrochemical plants) in 2001[4]. The mean T-PAH concentrations in industrial areas of the 15 cities/provinces in Korea measured as the sum fractions in the present study (4.27 ng $m^{-3}$) hence appear to fall in the intermediate range between various industrial sites across different countries selected for this comparison.

Although the concentration levels of T-PAHs in most industrial areas were quite high, these values were still lower than those of urban areas affected directly by mobile sources. In an area with high traffic volume (e.g., more than 170,000 vehicles per day), such as the urban area of Hunghom, Hong Kong (China), the T-PAH concentration in PM10 fraction was observed as 30.8 ng $m^{-3}$ during 2000–2001[9]. Similarly, at an urban monitoring station of Tunghai University, located in Taichung City, Taiwan, the T-PAH concentration in TSP fraction was found as 16.4 ng $m^{-3}$ during 2002–2003[21]. Relatively enhanced PAH levels at this site were ascribed to the single (or combined) effects of combustion (hospital incinerator) and/or vehicular sources (main road: in Taichung City)[21].

A comparison of the T-PAH concentration levels in relatively clean areas (e.g., suburban, rural, and greenland areas) confirms that the T-PAH concentrations in most Asian countries are significantly higher than those of the European and North American countries. For instance, the T-PAH concentrations of 8.58 ng $m^{-3}$ (sum phase) observed at a suburban area in Bangkok, Thailand in 2002[22] were significantly higher than those of a suburban area at Menen, Belgium (4.21 ng $m^{-3}$) in PM2.5 fraction[6]. If the T-PAH concentrations in rural and greenland areas in Asian countries are concerned, significantly large values are also observed, as shown in this study, e.g., 2.60 (Korea) and 3.09 ng $m^{-3}$ (Korea), respectively. Likewise, the measurements made at the University of Malaya (Kuala Lumpur, Malaysia) showed a T-PAH value of 1.33 ng $m^{-3}$ in PM10 fraction[23]. In contrast, the T-PAH levels (ng $m^{-3}$) of PM2.5 fraction measured in European and North American countries were 0.59 at Wingene, Belgium in 2002[4] and 0.09 at Galveston/Houston (Texas) during 1997–1998[11]. The high PAH concentration levels in the background areas of Asian countries were hence explained as the consequence of poor control efforts on the anthropogenic source processes, accompanied by the massive consumption of fossil fuels (e.g., a heating fuel for residential facilities). The results of this comparison thus suggest that a great deal of effort is required to control of PAH emissions in Asian countries.

## CONCLUSIONS

The data sets of seven PAH compounds in sum (gas + particle) phase were measured from 32 monitoring stations in 15 cities/provinces in Korea during a 2-year period (2006–2008). The data of each PAH compound

**TABLE 7**
**Comparison the PAH Concentration Data Measured in Industrial, Urban, and Background Areas on the Globe**

| Ref. No. | Site | City (Province)/ Country | Study Period | Fraction Size | Sample size (n) | BaA | CHRY | BbF | BkF | DahA | I123P | BaP | T-PAH | References |
|---|---|---|---|---|---|---|---|---|---|---|---|---|---|---|
| | | | | | | ($ng\ m^{-3}$) | | | | | | | | |
| *I. Industrial area* | | | | | | | | | | | | | | |
| 1[1] | Industrial area | Korea | Feb. 2006 – Jan. 2008 | TSP + Gas | | 0.57 | 0.79 | 1.06 | 0.65 | 0.3 | 0.42 | 0.47 | 4.27 | This study |
| 2 | Taichung industrial park | Taichung, Taiwan | Aug. – Dec. 2002 | TSP | 12 | 0.4 | 2.4 | 2.7 | 2.1 | 2.6 | 2.6 | 0.7 | 13.5 | Fang et al.[20] |
| 3 | Taichung industrial park | Taichung, Taiwan | Aug. – Dec. 2002 | Gas | 12 | 13.8 | 48.3 | 10.1 | 12.4 | 1.7 | 1.3 | 8.3 | 95.9 | Fang et al.[20] |
| 4 | Taichung industrial park | Taichung, Taiwan | 2002–2003 | TSP | 23 | 0.8 | 1.7 | 1.8 | 1.7 | 1.9 | 1.9 | 0.9 | 10.7 | Fang et al.[21] |
| 5 | Taichung industrial park | Taichung, Taiwan | 2002–2003 | Gas | 23 | 16.1 | 35.6 | 7.3 | 10.9 | 3.7 | 3.3 | 8.7 | 85.6 | Fang et al.[21] |
| 6[2] | Petroleumkaai | Flanders, Belgium | Sep. – Oct., 2001 | Particle + Gas | | 0.09 | 0.17 | 0.21 | 0.1 | 0.16 | 0.17 | 0.06 | 0.96 | Ravindra et al.[4] |
| 7[2] | Zelzate | Flanders, Belgium | Dec. 2001 – Jan. 2002 | Particle + Gas | | 0.65 | 1.87 | 1.73 | 0.9 | 0.55 | 1.76 | 1.27 | 8.73 | Ravindra et al.[4] |
| 8 | Kwun Tong | Hong Kong, China | Nov. 2000 – Aug. 2001 | PM10 | 31 | 0.6 | 1.95 | 5.2 | 0.29 | 3.22 | 1.91 | 1.3 | 14.5 | Guo et al.[9] |
| 9 | Clinton | Houston, Texas, USA | Mar. – June 1997 | PM2.5 | 12 | 1.29 | 1.34 | 0.97 | 0.87 | 0.9 | 0.96 | 0.87 | 7.20 | Fraser et al.[11] |
| 10 | Clinton | Houston, Texas, USA | July – Oct. 1997 | PM2.5 | 11 | 0.15 | 0.23 | 0.19 | 0.14 | 0.09 | 0.1 | 0.05 | 0.95 | Fraser et al.[11] |
| 11 | Clinton | Houston, Texas, USA | Nov. 1997 – Feb. 1998 | PM2.5 | 16 | 0.24 | 0.34 | 0.36 | 0.46 | 0.23 | 0.55 | 0.27 | 2.45 | Fraser et al.[11] |
| 12 | Elfsina, greater Athens area | Athens, Greek | Nov. 2001 – June 2002 | PM10 | | 0.299 | 0.444 | 0.968 | 0.435 | 0.222 | 1.001 | 0.714 | 4.08 | Mantis et al.[24] |
| *II. Urban area* | | | | | | | | | | | | | | |
| 13[1] | Residential area | Korea | Feb. 2006 – Jan. 2008 | TSP + Gas | | 0.48 | 0.77 | 1.23 | 0.54 | 0.47 | 0.41 | 0.51 | 4.42 | This study |
| 14[1] | Commercial area | Korea | Feb. 2006 – Jan. 2008 | TSP + Gas | | 0.49 | 1.04 | 1.47 | 0.61 | 0.21 | 0.4 | 0.62 | 4.85 | This study |
| 15 | National Chung-Hsing Uni. | Taichung, Taiwan | Aug. – Dec. 2002 | TSP | 12 | 1.1 | 2.1 | 2.4 | 2.3 | 1.8 | 2.4 | 0.9 | 13.0 | Fang et al.[20] |
| 16 | Tunghai University | Taichung, Taiwan | 2002–2003 | TSP | 23 | 0.9 | 2.2 | 1.5 | 3.2 | 5.2 | 1.9 | 1.5 | 16.4 | Fang et al.[21] |
| 17 | Dongdo school | Seoul, Korea | 1998–1999 | Particle + Gas | | 1.82 | 2.78 | | | 0.55 | 3.59 | 2.55 | 11.3 | Park et al.[25] |
| 18[2] | Borgerhout | Flanders, Belgium | Nov. – Dec. 2001 | Particle + Gas | | 0.26 | 0.91 | 0.9 | 0.49 | 0.39 | 0.31 | 0.78 | 4.04 | Ravindra et al.[4] |
| 19 | Hunghom | Hong Kong, China | Nov. 2000 – Aug. 2001 | PM10 | 30 | 1.06 | 3.4 | 10.9 | 0.4 | 7.99 | 4.93 | 2.13 | 30.8 | Guo et al.[9] |
| 20 | Rome | Italy | Mar. 1993 – Feb. 1998 | PM10 | | 0.82 | | | | 0.22 | 1.51 | 1.38 | 3.93 | Menichini et al.[26] |
| 21 | Nantou Township | Taiwan | Aug. 2008 | Gas | | 8.3 | 15.4 | 12.1 | 7.2 | | | 1.5 | 44.5 | Rau et al.[27] |
| 22 | Aristolelous, greater Athens area | Athens, Greek | 2001–2002 | PM10 | | 0.23 | 0.322 | 0.847 | 0.322 | 0.187 | 1.076 | 0.565 | 3.55 | Mantis et al.[4] |
| *III. Background area* | | | | | | | | | | | | | | |
| 23[1] | Background area | Korea | Feb. 2006 – Jan. 2008 | TSP + Gas | | 0.21 | 0.49 | 0.79 | 0.42 | 0.17 | 0.22 | 0.3 | 2.60 | This study |
| 24[1] | Greenland area | Korea | Feb. 2006 – Jan. 2008 | TSP + Gas | | 0.4 | 0.69 | 0.81 | 0.38 | 0.17 | 0.21 | 0.43 | 3.09 | This study |
| 25 | University of Malaya | Kuala Lumper, Malaysia | Mar. – Dec. 2001 | PM10 | 19 | | 0.07 | 0.25 | 0.38 | | 0.47 | 0.16 | 1.33 | Omar et al.[23] |
| 26[3] | Meteorological station, AIT | Bangkok, Thailand | 1996, 1997 | Particle + Gas | | 3.43 | 0.46 | 1.11 | 0.46 | 1.31 | 1.46 | 0.35 | 8.58 | Oanh et al.[22] |
| 27 | Jinàma Valley | Gran Canaria | 1995 | TSP | 96 | 0.103 | 0.046 | | | | | 0.129 | 0.28 | Cancio et al.[28] |
| 28 | Wingene | Flanders, Belgium | Apr. – May 2002 | Particle + Gas | | 0.08 | 0.09 | 0.17 | 0.06 | 0.05 | 0.09 | 0.05 | 0.59 | Ravindra et al.[4] |
| 29[3] | Menen | Belgium | 2003 | TSP | | 0.42 | 0.89 | 0.85 | 0.38 | 0.28 | 0.79 | 0.6 | 4.21 | Ravindra et al.[6] |
| 30 | Galveston/Houston | Texas, USA | Mar. 1997 – Feb. 1998 | PM2.5 | 15 | 0.01 | 0.02 | 0.02 | 0.02 | 0.01 | 0.01 | | 0.09 | Fraser et al.[11] |

1 The mean PAH concentration at each land use type are measured at 32 monitoring stations from 15 cities/provinces in Korea.

2 Sample size is seven to nine samples.

3 Suburban area.

and T-PAHs from all stations were used to describe their environmental behavior from various perspectives. Comparison of the individual PAHs showed that the high mean PAH levels occurred most frequently from CHRY and/or BbF. In contrast, low PAH values typically occurred from DahA in most stations. Moreover, the analysis of the T-PAH data between different cities/provinces also clearly indicated that those with strong manmade activities are characterized by notably high T-PAH concentration levels (IC, GG, and GB) as well as the cities of high population densities (SL and DG). In contrast, the clean cities/provinces surrounded by large suburban or rural areas (DJ, GJ, US, GW, GN, JN, JB, CN, CB, and BS) were characterized by low T-PAH levels, which ranged from 0.56 to 2.83 ng $m^{-3}$. When the concentrations of T-PAH were compared between different land use types, T-PAH pollution was found in the order of C > R > I > G > B.

According to the evaluation of T-PAH concentrations between seasons, T-PAH levels were lower in summer than in other seasons due to strong and frequent precipitation and the related meteorological conditions. A comparison of the annual mean values of T-PAHs during the 2-year period indicated contrasting patterns among the sites. Decreasing trends were apparent in highly industrial areas (e.g., GB, GG, and DG), while increasing trends were more dominant in large cities (SL and BS) and those with the low T-PAH concentrations (GJ and JB). Results of the correlation analysis for each PAH compound among cities/provinces indicated that strong correlations (class III) were most abundant for compounds emitted from mobile and industrial sources (e.g., BbF, I123P, and BaP). In addition, strong correlations were found abundantly from cities with strong industrial activities (e.g., IC and US) and large cities (e.g., SL and BS). A comparison of the T-PAH concentration levels measured from various sites on the globe revealed that the T-PAH concentrations in many Asian countries were noticeably higher than those of the European and Western countries. This difference may be due to increasing energy consumption in conjunction with limitations in emission control systems for the Asian countries.

## ACKNOWLEDGMENTS

This study was supported by a National Research Foundation of Korea (NRF) grant funded by the Ministry of Education, Science and Technology (MEST) (No. 2009-0093848).
